# Editorial: Innovative approaches in drug discovery and development

**DOI:** 10.3389/fmedt.2023.1206088

**Published:** 2023-05-12

**Authors:** Qasem Ramadan, Diego Romano Perinelli, Laura Orian, Sara Baratchi

**Affiliations:** ^1^ College of Science & General Studies, Alfaisal University, Riyadh, Saudi Arabia; ^2^ School of Pharmacy, University of Camerino, Camerino, Italy; ^3^ Dipartimento di Scienze Chimiche, Università degli Studi di Padova, Padova, Italy; ^4^ Baker Hearth and Diabetes Institute, Melbourne, VIC, Australia

**Keywords:** drug discovery, innovation, pharmaceutical, *in vitro*, technology

**Editorial on the Research Topic** Innovative approaches in drug discovery and development

In December 2022, the US House of Representatives has passed the FDA Modernization Act of 2022, which eliminates the animal-testing mandate for drug development and replaces this strategy with 21st-century methods grounded on human biology. This historical policy development reboots our drug development paradigm and drives us into a new era of drug discovery. The transition from bench to bedside has been long, and the number of drug withdrawals has increased to historic highs. Therefore, large investments are being made in translational research to find innovative tools that accelerate the drug discovery processes. During the last few years, we have seen many technological advances across the pharmaceutical industry that show tremendous potential to push the frontiers of pharmaceutical research to new vistas. In particular, the fusion of many engineering disciplines into the medical and pharmaceutical industries allows using the engineering principles to solve problems in biology and medicine ushering in a new era of technological breakthroughs for sensing and manipulating molecules, cells, tissues, and organs.

This Research Topic includes four papers that address the current innovation at the frontiers of pharmaceutical research and development.

Achieving enhanced drug delivery, efficacy and reduced toxicity effects of existing and new drug entities are crucial objectives in the pharmaceutical and medical device industries. The current trend in cardiovascular disease management has evolved significantly due to the improvement in both surgical and percutaneous revascularization techniques, which became the preferred therapeutic strategy in many clinical subgroups. To date, animal studies remain to play a vital role in validating the safety and efficacy of medical devices and biomaterials during the bench-to-clinic translation stage. However, the results obtained from these studies can be sometimes inconsistent and may fail in human studies.

Therefore, there is a real need for human-based *in vitro* or *ex vivo* models to accelerate the translation to the clinical stage. For instance, the evaluation of non-stent cardiovascular drug delivery systems is lacking *ex vivo* models capable of testing pharmacokinetic performance in humans. Cooper et al. developed an *ex vivo* system to quantify the acute drug transfer of catheter-based drug delivery devices (a paclitaxel-coated balloon and a perfusion catheter) into explanted carotid arteries. To validate the *ex vivo* measurements, parallel experiments were also performed in a pig model. Overall, the results demonstrated no significant differences between *ex vivo* and *in vivo* outcomes for the two tested drug delivery devices. This system would reduce the time and expense associated with *in vivo* testing of vascular devices, particularly in measuring and quantifying vessel drug retention.

Besides *ex vivo* and *in vitro* models, computer-aided drug design (CADD) and *in silico* methodologies are increasingly used in various stages of drug discovery and development which significantly reduce the costs and time. Verma et al. used CADD to investigate the potential antifungal effect of the 1, 2, 4-triazine and its derivatives and if it can protect humans from infection with *Candida albicans* by employing the molecular docking and molecular dynamics simulation and aiming at inhibition of *Candida albicans* CYP51. It was found that each drug has a high binding affinity for CYP51 proteins and is involved in non-covalent interactions and hydrogen bonds with their active residues and surrounding allosteric residues which establish a strong candidature of 1, 2, 4-triazine and its derivatives as a potential drug target against fungal infection.

Organ-on-a-chip has emerged as a promising technology to provide reliable *in vitro* tools for solving key issues in drug discovery and bridging the gap between *in vivo* and *in vitro* studies. These humanized microphysiological models have shown rapid progress during the last few years as indispensable tools for recapitulating human physiological key-parameters, hence enabling the translation of the preclinical findings at the Pre-Investigational New Drug Application (Pre-IND) stage. In his opinion paper, Nahle provides a dozen reasons why organ-on-a-chip systems are better at modeling human physiology, drug-organ interaction and diseases. The paper highlights the key-findings of the recent study by Ewart et al. (1) and comments on the implication of the work in the context of the growing demand for human relevancy across the preclinical stages of drug development.

Finally, the study by Khalil and Onyango discusses the effect of patent expiry on the performance of innovator multinational pharmaceutical companies in a low-middle income country, such as Kenya. The study focuses on the effect of generic products manufacturing and competitive market pressures, price changes, and changes in sales volumes and profitability of innovator multinational pharmaceutical companies after patent expiry. Qualitative and quantitative techniques were used to collect relevant data via survey questionnaires and in-depth interviews with regional managers, general managers, and directors of eight participating companies. The results of the study depicted a significant effect of patent expiry on the generic production and subsequent decline in the performance of multinational innovator companies in the pharmaceutical industry. This study also recommends the multinational innovator companies operating in low-income countries to develop strategic policies that encourage collaborative manufacturing with generic companies to share revenues.

We will see more advanced technologies infuse into the lab practice over the next few years and more innovations will continue to emerge, and it is exciting to think how drug discovery will develop in the near future.
